# The impact of different ovarian stimulation protocols on the expression levels of GDF-9 and BMP-15 in cumulus cells of follicles

**DOI:** 10.3389/fendo.2025.1572388

**Published:** 2025-06-04

**Authors:** Lili Cheng, Jie Zhang, Dongxiu Li, Pengyu Xu, Shan Liu, Ruijuan Guo, Xue Wang, Li Zhang

**Affiliations:** ^1^ Department of Reproductive Medicine, Hebei Medical University Third Hospital, Shijiazhuang, Hebei, China; ^2^ Department of Reproductive Medicine, Bethune International Peace Hospital, Shijiazhuang, Hebei, China

**Keywords:** oocyte-secreted factors, granulosa cells, oocyte maturation, controlled ovarian stimulation, embryo development

## Abstract

**Objective:**

To analyze the expression levels of the oocyte-secreted factors growth differentiation factor-9 (GDF-9) and bone morphogenetic protein-15 (BMP-15) in cumulus cells (CCs) under different controlled ovarian stimulation (COS) protocols and their association with oocyte maturity and embryo developmental potential.

**Methods:**

This study included 76 patients requiring intracytoplasmic sperm injection (ICSI) due to severe oligoasthenoteratozoospermia or previous ICSI treatment, resulting in the collection of 749 CC samples. Patients were divided into four groups based on COS protocols: short-acting luteal phase (14 patients, 168 CCs), long-acting follicular phase (21 patients, 189 CCs), micro-stimulation (12 patients, 86 CCs) and antagonist (29 patients, 306 CCs). The mRNA was extracted from cumulus granulosa cells, and the relative levels of GDF-9 and BMP-15 were measured using real-time quantitative PCR (Q-PCR). The expression levels of GDF-9 and BMP-15 were compared across different ovarian stimulation protocols, while oocyte maturation, fertilization, cleavage, and blastocyst formation were assessed. The expression levels of GDF-9 and BMP-15 were compared across protocols, and oocyte maturation, fertilization, cleavage and blastocyst formation were assessed.

**Results:**

GDF-9 and BMP-15 levels were substantially higher in MII oocytes than in MI and GV oocytes and were also elevated in the normal fertilization group, high-quality cleavage embryos and high-quality blastocysts. Growth differentiation factor-9 expression was higher in the short-acting luteal phase protocol than in the antagonist protocol, whereas BMP-15 expression was higher in both the short-acting luteal phase and long-acting follicular phase protocols compared with the micro-stimulation and antagonist groups.

**Conclusion:**

GDF-9 and BMP-15 are reliable indicators of oocyte developmental potential. The long-acting follicular phase and short-acting luteal phase protocols enhance oocyte maturity and embryo development, whereas the micro-stimulation and antagonist protocols appear less favorable.

## 1 Introduction

With advancements in society and increasing competitive pressures, many women are delaying childbirth, leading to a growing proportion of older couples seeking to conceive and a rising incidence of infertility (1). *In vitro* fertilization and embryo transfer (IVF-ET) has become a primary assisted reproductive technology, with treatment protocols gradually becoming more individualized to address various aetiologies (2). A key determinant of IVF success is the choice of controlled ovarian stimulation protocol, which substantially influences oocyte quality and embryo developmental potential (3).

Common ovarian stimulation protocols include the long-acting follicular phase protocol, short-acting luteal phase protocol, antagonist protocol and micro-stimulation protocol (4). Despite their widespread clinical application, the efficacy and suitability of these protocols remain debated due to the heterogeneity of patient populations and individual differences in response to stimulation (5). Although numerous clinical studies compare the therapeutic outcomes of these protocols, laboratory-based research specifically examining their impact on oocyte-secreted factor expression remains limited.

Oocyte-secreted factors (OSFs) play a crucial role in oocyte development, with growth differentiation factor-9 (GDF-9) and bone morphogenetic protein-15 (BMP-15) being key OSFs (6–8). These factors regulate granulosa cell proliferation, apoptosis and metabolism, thereby influencing oocyte maturation and quality, which ultimately determine embryo developmental potential (9–11). Although studies have demonstrated a close association between GDF-9 and BMP-15 expression and oocyte quality (12), limited research has investigated how different ovarian stimulation protocols affect their expression.

This study aims to examine the impact of various ovarian stimulation protocols on the expression levels of GDF-9 and BMP-15 in oocyte granulosa cells and to analyze the relationship between these factors, oocyte maturity and embryo developmental potential. By addressing this research gap, we aim to establish a theoretical foundation for optimizing ovarian stimulation protocols in IVF and provide more precise guidance for individualized treatment strategies.

## 2 Materials and methods

### 2.1 Study participants

This prospective cohort study was conducted on patients undergoing intracytoplasmic sperm injection (ICSI) treatment at Hebei Medical University Third Hospital and Bethune International Peace Hospital between January 2020 and June 2024. Eligible participants included those requiring ICSI due to severe oligoasthenoteratozoospermia in individuals with testes or a history of failed or low fertilization in IVF-ET. All patients received routine clinical treatment, and those who met the inclusion criteria but did not fulfill the exclusion criteria were enrolled for data collection.

**Figure 1:**
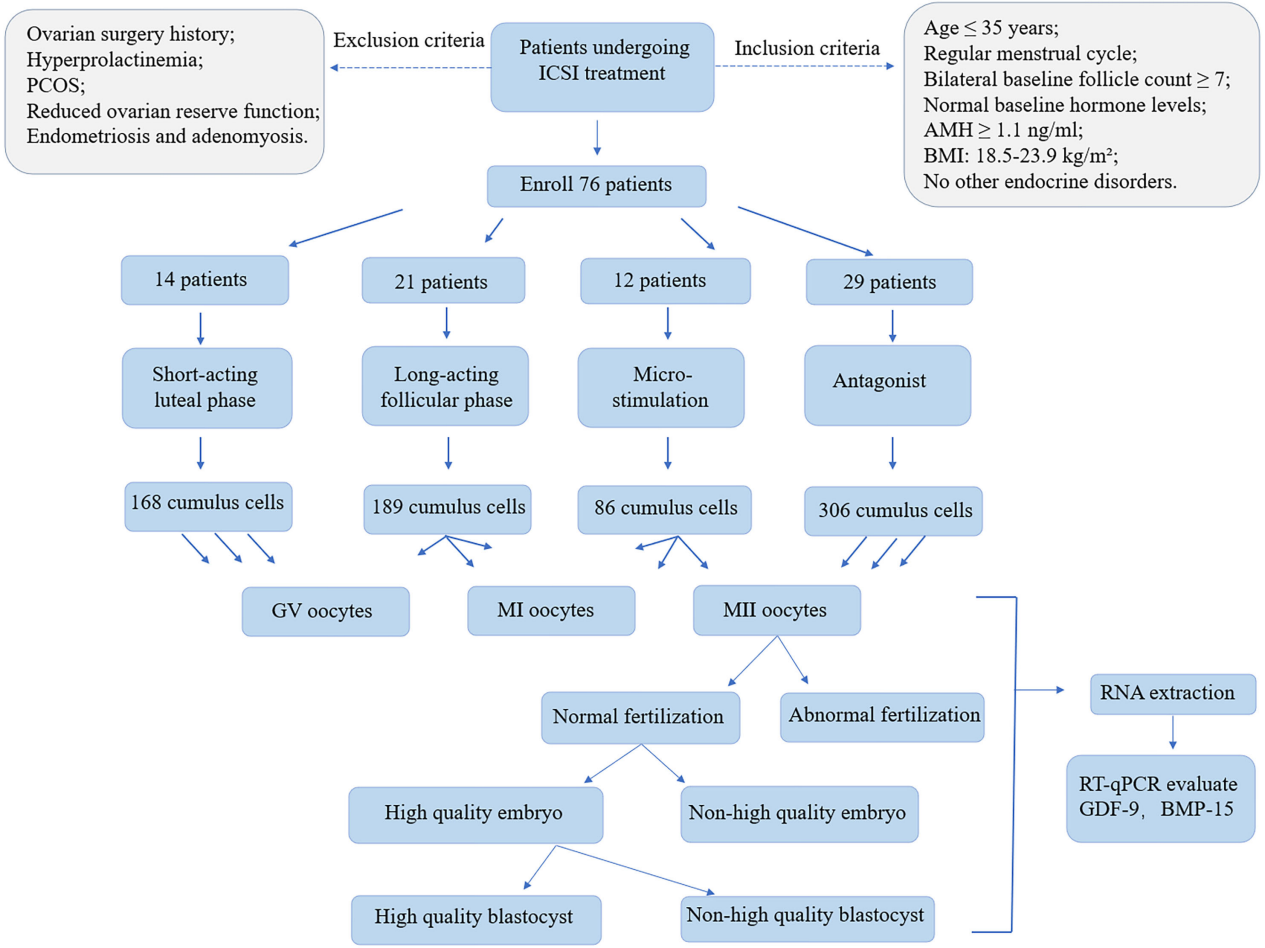
The study flow diagram. The whole design includes patient enrollment, cumulus cells collection, oocyte collection. RNA was extracted from both GV oocytes, MI oocytes, MII oocytes to evaluate GDF-9, BMP-15 expression levels. MII oocytes further divided into different group and RNA was extracted from all groups to evaluate GDF-9, BMP-15 expression levels.

Intracytoplasmic sperm injection technology enables the direct selection of a single sperm with normal morphology and high motility for injection into the oocyte, thereby minimizing the potential impact of sperm quality on embryonic development during natural fertilization (study flow shown in **Figure 1**).

The inclusion criteria were as follows: Age ≤ 35 years, regular menstrual cycle (28 ± 7 days), bilateral baseline follicle count ≥ 7, normal baseline hormone levels, anti-Müllerian hormone (AMH) ≥ 1.1 ng/ml, body mass index (BMI) between 18.5 and 23.9 kg/m² and no other endocrine disorders.

The exclusion criteria were as follows: History of ovarian surgery, hyperprolactinaemia, polycystic ovary syndrome, diminished ovarian reserve, endometriosis and adenomyosis.

This study was approved by the ethics committee of the Hebei Medical University Third Hospital (W2021-007-1).

### 2.2 Grouping method

Following standard clinical practices in IVF-ET and the European Society of Human Reproduction and Embryology (ESHRE) guidelines on ovarian stimulation for IVF (13), participants were categorized into four groups: Group A (short-term long protocol during the luteal phase), Group B (long-term long protocol during the follicular phase), Group C (micro-stimulation protocol) and Group D (antagonist protocol).

Based on oocyte maturation, cumulus cells (CCs) were classified into GV, MI and MII stages. The MII group was further divided into normal and abnormal fertilization groups. The normal fertilization group was subsequently categorized into high-quality and non-high-quality embryos based on Day 3 embryo scoring. Embryos were then cultured to assess blastocyst formation on Days 5 and 6 and classified into high-quality and non-high-quality blastocyst groups.

### 2.3 Controlled ovarian stimulation medication protocols

#### 2.3.1 Short-term GnRH-a long protocol (luteal phase)

Administer short-term GnRH-a (Dabigatran, Bayer, Germany) at a dosage of 0.1 mg daily, starting 7 days after ovulation or when five to seven contraceptive pills remain in the combined oral contraceptive cycle. After 16–18 days, initiate gonadotropin (Gn) (Gonal-f, Merck Serono, Switzerland) at 150–300 IU/day for ovulation induction once downregulation is confirmed. Trigger ovulation with 250 µg of hCG (Merck Serono, Italy) when follicles reach ≥18 mm (at least two follicles or ≥ three follicles measuring ≥17 mm). Oocyte retrieval is performed 36 hours later.

#### 2.3.2 Long-term GnRH-a long protocol (follicular phase)

Administer long-acting GnRH-a (leuprolide acetate, Shanghai Livzon Pharmaceutical) at a dosage of 3.75 mg on the 2nd or 3rd day of menstruation for downregulation. Initiate Gn at 150–300 IU/day for ovulation induction 28–40 days later. Trigger ovulation with 250 µg of hCG once follicular criteria are met, followed by oocyte retrieval 36 hours later.

#### 2.3.3 Micro-stimulation protocol

Administer letrozole (Jiangsu Hengrui) at 5 mg/day and human menopausal gonadotropin (Guangzhou Lizhu Group) at 75–150 IU/day, starting on the 2nd or 3rd day of menstruation. After 5 days, replace letrozole with clomiphene citrate (Cyprus Gout Pharmaceutical Co., Ltd) at 50 mg/day. Adjust the Gn dosage based on ovarian response. If LH levels reach ≥15 U/L, administer Cetrorelix (Cetrotide, Merck Serono) at 0.125–0.25 mg/day to suppress the LH surge. Trigger ovulation with 250 µg of hCG (Merck Serono, Italy) or a combination of 0.2 mg Triptorelin (Dabigatran, Bayer, Germany) and 2,000 IU of hCG (Injectable Chorionic Gonadotropin, Shanghai Lizhu Group), followed by oocyte retrieval.

#### 2.3.4 Antagonist protocol

Initiate recombinant human FSH at 150–300 IU/day on the 2nd or 3rd day of menstruation. Introduce cetrorelix (cetrotide, Merck Serono, Switzerland) at 0.25 mg/day when follicles reach 12–14 mm and/or E2 levels exceed 1,468 pmol/L, with LH >5 IU/L. Trigger ovulation with 250 µg of hCG (Merck Serono, Italy), followed by oocyte retrieval 36 hours later.

### 2.4 Collection of cumulus granulosa cell mass

Transvaginal oocyte retrieval under ultrasound guidance is performed 36 hours after the trigger. The cumulus-oocyte complex is placed in individual microdrops of embryo processing solution. Between 40 and 42 hours post-trigger, the CCs surrounding the oocyte are treated with hyaluronidase and mechanically denuded.

The remaining cumulus granulosa cells in the microdrop are transferred into small EP tubes, repeatedly washed with PBS (pH 7.4) and centrifuged. The PBS is then removed, leaving only the granulosa cells at the bottom of the tube. These cells are frozen at −80°C and labelled to ensure a one-to-one correspondence between the oocyte and the collected CCs.

### 2.5 Cumulus-oocyte complex maturity assessment, fertilization, cleavage stage and blastocyst grading

The expression levels of OSFs are observed in GV, MI and MII stage oocytes. All MII stage oocytes undergo ICSI fertilization, with continuous monitoring of fertilization, cleavage and blastocyst formation. Pronuclear assessment is performed 16–18 hours post-fertilization, where the presence of two pronuclei indicates normal fertilization, while 0, 1 or ≥3 pronuclei indicate abnormal fertilization. Intracytoplasmic sperm injection fertilization and culture in microdrops are conducted 40–42 hours post-trigger.

Embryo evaluation follows the ESHRE guidelines, with fertilization confirmed approximately 16–18 hours post-ICSI. Normal fertilization is indicated by the presence of 2PN, whereas other outcomes denote abnormal fertilization. Day 3 embryos are assessed using the Peter cleavage stage embryo scoring system (14), where high-quality embryos are defined as those with 6–10 blastomeres of equal or slightly unequal sizes and ≤20% fragmentation. The specific laboratory criteria for high-quality embryos include 7–9 blastomeres, regular arrangement and ≤20% fragmentation.

On Day 5, blastocysts are graded using the Gardner blastocyst scoring system (15, 16). High-quality blastocysts are classified as those with grades ≥4AA, 4AB, 4BA or 4BB.

### 2.6 RNA extraction and reverse transcription reaction

Cells at different developmental stages, including oocytes, embryos and blastocysts, are collected for RNA isolation. RNA extraction is performed using Trizol. RNA samples are treated with DNase I to degrade contaminating DNA. Extracted RNA is assessed for quality using agarose gel electrophoresis, followed by reverse transcription (RT) according to the instructions provided with the Reverse Transcription Reaction kit (Promega Biotech Co., Ltd.). The RT reaction is carried out under the following conditions: 70°C for 10 minutes, 42°C for 50 minutes and 95°C for 5 minutes.

### 2.7 Real-time fluorescent quantitative polymerase chain reaction

*RPL-15* is used as the reference gene due to its stable expression in CCs under experimental conditions. Primers for the target genes (*GDF-9 and BMP-15*) and the reference gene (*RPL-15*) are designed using PrimerPremier 5.0 software (see **Table 1**). Primers with optimal melting temperatures (Tm) and minimal secondary structures are selected for amplification. The reaction is conducted following the instructions of the QuantiFast^®^ SYBR^®^ Green PCR kit. To ensure PCR quality control, no-template controls are included to detect non-specific amplification, whereas no-RT controls are used to identify DNA contamination. Each sample is run in three technical replicates to minimize variability. The mean value of duplicate runs is used for statistical analysis.

**Table 1:** Primer sequence.

Gene	Primer sequence	Size (bp)
GDF-9	Forward 5’-TTCTATCTGTTGGGCGAGGT-3’	250
	Reverse 5’-TCAACGGTAGTAATGCGATCC-3’	
BMP-15	Forward 5’-TAGAGAGAACCGCACCATTG-3’	187
	Reverse 5’- GAAGCGAGTTAGTTGGAGATGAT-3’	
RPL-15	Forward 5’-GACCCCAATGAGACCAATGAAATC-3’	105
	Reverse 5’-GGAATGGACCGTCACAGGCTTG-3’	

### 2.8 Statistical methods

The relative expression levels of GDF-9 and BMP-15 RNA in each group are analyzed using real-time PCR. The obtained Ct values are calculated using the formula 2ΔCt (17), where ΔCt = Ct (target gene) − Ct (reference gene).

Statistical analyses are conducted using SPSS 25.0 (IBM, Armonk, NY, USA). Normally distributed data are presented as mean ± standard deviation ( $\bar{x}$ ± s) and compared using independent sample t-tests. Non-normally distributed data are expressed as median (P25, P75) and analyzed using Mann-Whitney U tests, with P < 0.05 indicating statistical significance. Comparisons between different protocol groups are performed using F-tests, with P < 0.05 considered statistically significant.

## 3 Results

### 3.1 Comparison of general conditions among the four groups

There were no statistically significant differences in age, infertility duration, BMI, AMH levels or baseline hormone levels among the four groups (all P > 0.05), indicating comparability between groups. Although no significant differences were observed in the number of Gn days, there were significant differences in total Gn dosage. The microstimulation protocol required a significantly lower Gn dose than the other three groups (P = 0.000), whereas no significant differences were found among the remaining three groups (**Table 2**).

**Table 2:** Comparison of basic information in four group ( $\bar{x}$ ± s).

	A group (n=14)	B group (n=21)	C group (n=12)	D group (n=12)	F	P
Age (year)	29.57 ± 3.16	30.71 ± 2.80	31.00 ± 2.56	29.45 ± 2.64	1.467	0.231
Duration of infertility	3.79 ± 1.58	3.38 ± 1.53	3.42 ± 1.78	3.24 ± 1.35	0.409	0.747
BMI (kg/m2)	21.69 ± 1.60	22.11 ± 1.17	21.00 ± 1.40	22.05 ± 1.11	2.421	0.073
AFC	12.36 ± 1.78	11.81 ± 3.01	10.75 ± 1.76	12.21 ± 2.16	1.344	0.267
bFSH (mIU/ml)	6.22 ± 1.00	5.89 ± 1.12	5.64 ± 0.97	6.00 ± 1.16	0.647	0.588
bE2 (pg/ml)	35.83 ± 8.75	40.46 ± 12.16	35.20 ± 9.05	38.12 ± 14.39	0.647	0.588
Gn used days (d)	10.57 ± 1.22	10.38 ± 1.02	10.42 ± 1.08	10.28 ± 1.19	0.217	0.884
Gn used dose	2096.07 ± 136.74a	2055.95 ± 135.07a	1856.25 ± 202.30	2105.86 ± 133.58a	8.803	0.000

Group A: short-term long protocol during the luteal phase, Group B: long-term long protocol during the follicular phase, Group C: micro-stimulation protocol, and Group D: antagonist protocol.

### 3.2 Relationship between the relative expression of GDF-9 in CCs and oocyte maturity and developmental potential

As shown in **Tables 3**–**6**, across the four ovarian stimulation protocols, the relative expression levels of GDF-9 were significantly higher in MII oocytes compared with MI oocytes (fold change > 2, P = 0.037; P = 0.029; P = 0.016). Additionally, expression levels in MI oocytes were significantly higher than in GV oocytes (fold change > 1.5, P = 0.046; P = 0.014; P = 0.026).

**Table 3:** Expression levels of GDF-9 and BMP-15 in the short-term GnRH-a long protocol during the luteal phase ( $\bar{x}$ ± s).

Group	n (168)	GDF-9	P Value (GDF-9)	BMP-15	P Value (BMP-15)
GV	12	0.010 ± 0.002	GV vs MI: P=0.046, GV vs MII: P=0.029	0.113 ± 0.027	GV vs MI: P=0.046, GV vs MII: P=0.025
MI	28	0.011 ± 0.002	MI vs MII: P=0.037, MI vs Normal fertilization: P=0.028	0.112 ± 0.031	MI vs Normal fertilization: P=0.030
MII	128	0.013 ± 0.003	MII vs Abnormal fertilization: P=0.036, MII vs High-quality embryo: P=0.007	0.149 ± 0.035	MII vs Abnormal fertilization: P=0.023, MII vs High-quality blastocyst: P=0.016
Normal fertilization	112	0.013 ± 0.003	Normal fertilization vs Poor-quality blastocyst: P=0.048	0.147 ± 0.031	Normal fertilization vs Poor-quality embryo: P=0.014
Abormal fertilization	16	0.010 ± 0.001	Abnormal fertilization vs Poor-quality embryo formation: P=0.014	0.118 ± 0.030	Abnormal fertilization vs Poor-quality blastocyst: P=0.038
Good-quality embryo formation	45	0.014 ± 0.004	Good-quality embryo vs Poor-quality embryo: P=0.014	0.167 ± 0.046	Good-quality embryo vs Poor-quality blastocyst: P=0.029
Poor-quality embryo formation	67	0.013 ± 0.003	Poor-quality embryo vs Good-quality blastocyst: P=0.029	0.145 ± 0.032	Poor-quality embryo vs High-quality embryo: P=0.035
Good-quality blastocyst formation	19	0.015 ± 0.003	Good-quality blastocyst vs Poor-quality blastocyst: P=0.037	0.171 ± 0.023	Good-quality blastocyst vs Poor-quality embryo: P=0.028
Poor-quality blastocyst formation	52	0.013 ± 0.002		0.137 ± 0.0331	Poor-quality blastocyst vs High-quality blastocyst: P=0.038

**Table 4:** Expression levels of GDF-9 and BMP-15 in the long-term GnRH-a Long protocol during the follicular phase ( $\bar{x}$ ± s).

Group	n (189)	GDF-9	P Value (GDF-9)	BMP-15	P Value (BMP-15)
GV	12	0.012 ± 0.002	GV vs MI: P=0.046, GV vs MII: P=0.029	0.127 ± 0.022	GV vs MI: P=0.039, GV vs MII: P=0.033
MI	35	0.012 ± 0.002	MI vs MII: P=0.037, MI vs Normal fertilization: P=0.028	0.132 ± 0.030	MI vs Normal fertilization: P=0.025
MII	142	0.013 ± 0.003	MII vs Abnormal fertilization: P=0.036, MII vs High-quality embryo: P=0.008	0.149 ± 0.041	MII vs High-quality embryo: P=0.012, MII vs Poor-quality blastocyst: P=0.024
Normal fertilization	121	0.014 ± 0.004	Normal fertilization vs Poor-quality blastocyst: P=0.048	0.153 ± 0.043	Normal fertilization vs Poor-quality embryo: P=0.019
Abormal fertilization	21	0.012 ± 0.003	Abnormal fertilization vs Poor-quality embryo formation: P=0.014	0.130 ± 0.024	Abnormal fertilization vs Poor-quality blastocyst: P=0.038
Good-quality embryo formation	52	0.014 ± 0.004	Good-quality embryo vs Poor-quality embryo: P=0.014	0.161 ± 0.048	Good-quality embryo vs Poor-quality blastocyst: P=0.033
Poor-quality embryo formation	69	0.013 ± 0.003	Poor-quality embryo vs Good-quality blastocyst: P=0.029	0.146 ± 0.037	Poor-quality embryo vs High-quality embryo: P=0.028
Good-quality blastocyst formation	24	0.016 ± 0.003	Good-quality blastocyst vs Poor-quality blastocyst: P=0.037	0.153 ± 0.046	Good-quality blastocyst vs Poor-quality embryo: P=0.036
Poor-quality blastocyst formation	57	0.012 ± 0.002		0.147 ± 0.031	Poor-quality blastocyst vs High-quality blastocyst: P=0.044

**Table 5:** Expression levels of GDF-9 and BMP-15 in the mild stimulation protocol ( $\bar{x}$ ± s).

Group	n (86)	GDF-9	P Value (GDF-9)	BMP-15	P Value (BMP-15)
GV	6	0.012 ± 0.002	GV vs MI: P=0.040, GV vs MII: P=0.029	0.115 ± 0.025	GV vs MI: P=0.042, GV vs MII: P=0.038
MI	16	0.012 ± 0.002	MI vs MII: P=0.035, MI vs Normal fertilization: P=0.026	0.112 ± 0.032	MI vs Normal fertilization: P=0.032
MII	64	0.013 ± 0.004	MII vs Abnormal fertilization: P=0.028, MII vs High-quality embryo: P=0.010	0.131 ± 0.031	MII vs High-quality embryo: P=0.015, MII vs Poor-quality blastocyst: P=0.031
Normal fertilization	52	0.013 ± 0.004	Normal fertilization vs Poor-quality blastocyst: P=0.038	0.135 ± 0.031	Normal fertilization vs Poor-quality embryo: P=0.025
Abormal fertilization	12	0.012 ± 0.003	Abnormal fertilization vs Poor-quality embryo formation: P=0.042	0.115 ± 0.020	Abnormal fertilization vs Poor-quality blastocyst: P=0.029
Good-quality embryo formation	16	0.015 ± 0.005	Good-quality embryo vs Poor-quality embryo: P=0.023	0.145 ± 0.040	Good-quality embryo vs Poor-quality blastocyst: P=0.031
Poor-quality embryo formation	36	0.013 ± 0.003	Poor-quality embryo vs Good-quality blastocyst: P=0.027	0.131 ± 0.026	Poor-quality embryo vs High-quality embryo: P=0.031
Good-quality blastocyst formation	8	0.015 ± 0.002	Good-quality blastocyst vs Poor-quality blastocyst: P=0.035	0.154 ± 0.033	Good-quality blastocyst vs Poor-quality embryo: P=0.027
Poor-quality blastocyst formation	28	0.012 ± 0.003		0.125 ± 0.021	Poor-quality blastocyst vs High-quality blastocyst: P=0.031

**Table 6:** Expression levels of GDF-9 and BMP-15 in the antagonist protocol ( $\bar{x}$ ± s).

Group	n (306)	GDF-9	P Value (GDF-9)	BMP-15	P Value (BMP-15)
GV	19	0.011 ± 0.002	GV vs MI: P=0.042, GV vs MII: P=0.036	0.106 ± 0.025	GV vs MI: P=0.028, GV vs MII: P=0.022
MI	55	0.012 ± 0.003	MI vs MII: P=0.027, MI vs Normal fertilization: P=0.025	0.107 ± 0.027	MI vs Normal fertilization: P=0.032
MII	232	0.014 ± 0.003	MII vs Abnormal fertilization: P=0.023, MII vs High-quality embryo: P=0.011	0.136 ± 0.032	MII vs High-quality embryo: P=0.014, MII vs Poor-quality blastocyst: P=0.026
Normal fertilization	181	0.014 ± 0.004	Normal fertilization vs Poor-quality blastocyst: P=0.033	0.139 ± 0.033	Normal fertilization vs Poor-quality embryo: P=0.029
Abormal fertilization	51	0.012 ± 0.003	Abnormal fertilization vs Poor-quality embryo formation: P=0.037	0.125 ± 0.027	Abnormal fertilization vs Poor-quality blastocyst: P=0.028
Good-quality embryo formation	68	0.015 ± 0.004	Good-quality embryo vs Poor-quality embryo: P=0.017	0.146 ± 0.036	Good-quality embryo vs Poor-quality blastocyst: P=0.032
Poor-quality embryo formation	113	0.013 ± 0.003	Poor-quality embryo vs Good-quality blastocyst: P=0.028	0.135 ± 0.031	Poor-quality embryo vs High-quality embryo: P=0.025
Good-quality blastocyst formation	28	0.015 ± 0.003	Good-quality blastocyst vs Poor-quality blastocyst: P=0.024	0.157 ± 0.026	Good-quality blastocyst vs Poor-quality embryo: P=0.021
Poor-quality blastocyst formation	93	0.013 ± 0.003		0.134 ± 0.032	Poor-quality blastocyst vs High-quality blastocyst: P=0.029

Significant differences in GDF-9 expression were observed between the normal and abnormal fertilization groups (fold change > 1.5, P = 0.001; P = 0.036; P = 0.028). Expression levels in high-quality embryo groups were significantly higher than in non-high-quality embryo groups (fold change > 1.8, P = 0.007; P = 0.014; P = 0.026). Similarly, expression levels in high-quality blastocyst groups were significantly higher than in non-high-quality blastocyst groups (fold change > 2, P = 0.048; P = 0.029; P = 0.016).

### 3.3 Relationship between the relative expression of BMP-15 in CCs and oocyte maturity and developmental potential

Across the four ovarian stimulation protocols, the relative expression of BMP-15 in MII oocytes was significantly higher than in MI oocytes (P = 0.046; P = 0.025; P = 0.049) and expression in MI oocytes was significantly higher than in GV oocytes (P = 0.000; P = 0.038; P = 0.044). Expression levels in normal fertilization groups were significantly higher than in abnormal fertilization groups (P = 0.012; P = 0.034; P = 0.024). Additionally, expression levels in high-quality embryo formation groups were significantly higher than in non-high-quality embryo groups (P = 0.015; P = 0.002; P = 0.040). Statistically significant differences in BMP-15 expression were also observed between high-quality blastocyst groups and non-high-quality blastocyst groups (P = 0.040; P = 0.049; P = 0.037).

### 3.4 Comparison of relative expression levels of GDF-9 and BMP-15 in four ovulation induction protocols

There were no significant statistical differences in general clinical data among the four ovulation induction protocols: luteal phase standard long protocol, follicular phase long protocol, microstimulation protocol and antagonist protocol. However, Gn amounts were significantly lower in the microstimulation protocol compared with the luteal phase long protocol, follicular phase long protocol and antagonist protocol (P = 0.000).

**Figure 2:**
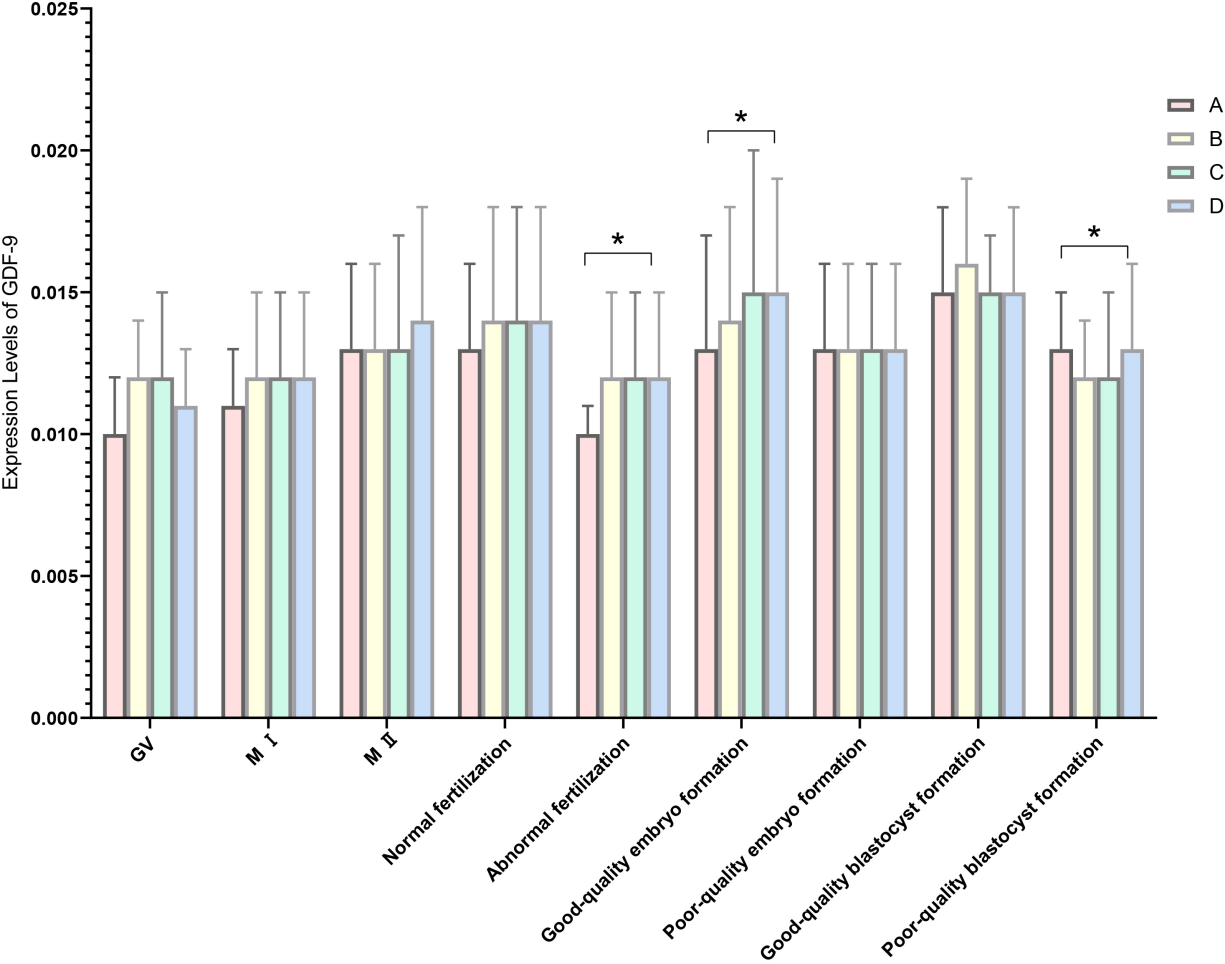
Expression levels of GDF-9 in four ovarian stimulation protocols. The different ovarian stimulation regiments are represented by different colored column. Group A is short-term long protocol during the luteal phase, displayed in red column; Group B is long-term long protocol during the follicular phase, displayed in yellow column; Group C is micro-stimulation protocol, displayed in green column; Group D is antagonist protocol, displayed in blue column. The Y-axis represents the mRNA expression level of GDF-9 obtained by RT-qpcr, n=3, * indicates p < 0.05. The X-axis indicates the different stages of RT-qpcr detection.

There were statistically significant differences in the relative expression of GDF-9 during the fertilization stage between the luteal phase standard long protocol and antagonist protocol (P = 0.007) and during the cleavage stage between the luteal phase standard long protocol and antagonist protocol (P = 0.002). Statistically significant differences were also observed during the blastocyst formation stage between the microstimulation protocol and antagonist protocol (P = 0.038) (**Figure 2**, **Table 7**).

**Table 7:** Expression levels of GDF-9 in four ovarian stimulation protocols ( $\bar{x}$ ± s).

Group	A group	B group	C group	D group	P Value
GV	0.010 ± 0.002	0.012 ± 0.002	0.012 ± 0.003	0.011 ± 0.002	0.076
MI	0.011 ± 0.002	0.012 ± 0.003	0.012 ± 0.003	0.012 ± 0.003	0.463
MII	0.013 ± 0.003	0.013 ± 0.003	0.013 ± 0.004	0.014 ± 0.004	0.079
Normal fertilization	0.013 ± 0.003	0.014 ± 0.004	0.014 ± 0.004	0.014 ± 0.004	0.115
Abormal fertilization	0.010 ± 0.001^a^	0.012 ± 0.003	0.012 ± 0.003	0.012 ± 0.003	0.025
Good-quality embryo formation	0.013 ± 0.004^a^	0.014 ± 0.004	0.015 ± 0.005	0.015 ± 0.004	0.014
Poor-quality embryo formation	0.013 ± 0.003	0.013 ± 0.003	0.013 ± 0.003	0.013 ± 0.003	0.689
Good-quality blastocyst formation	0.015 ± 0.003	0.016 ± 0.003	0.015 ± 0.002	0.015 ± 0.003	0.641
Poor-quality blastocyst formation	0.013 ± 0.002	0.012 ± 0.002	0.012 ± 0.003^a^	0.013 ± 0.003	0.048

^a^indicates a statistical difference compared to group D.

The relative expression of BMP-15 in MI oocytes was significantly different in the follicular phase long protocol compared with the other three groups (P = 0.011, P = 0.030, P = 0.000). In MII oocytes, expression levels were significantly different in the luteal phase standard long protocol compared with the follicular phase long protocol and microstimulation protocol (P = 0.033, P = 0.043).

**Figure 3:**
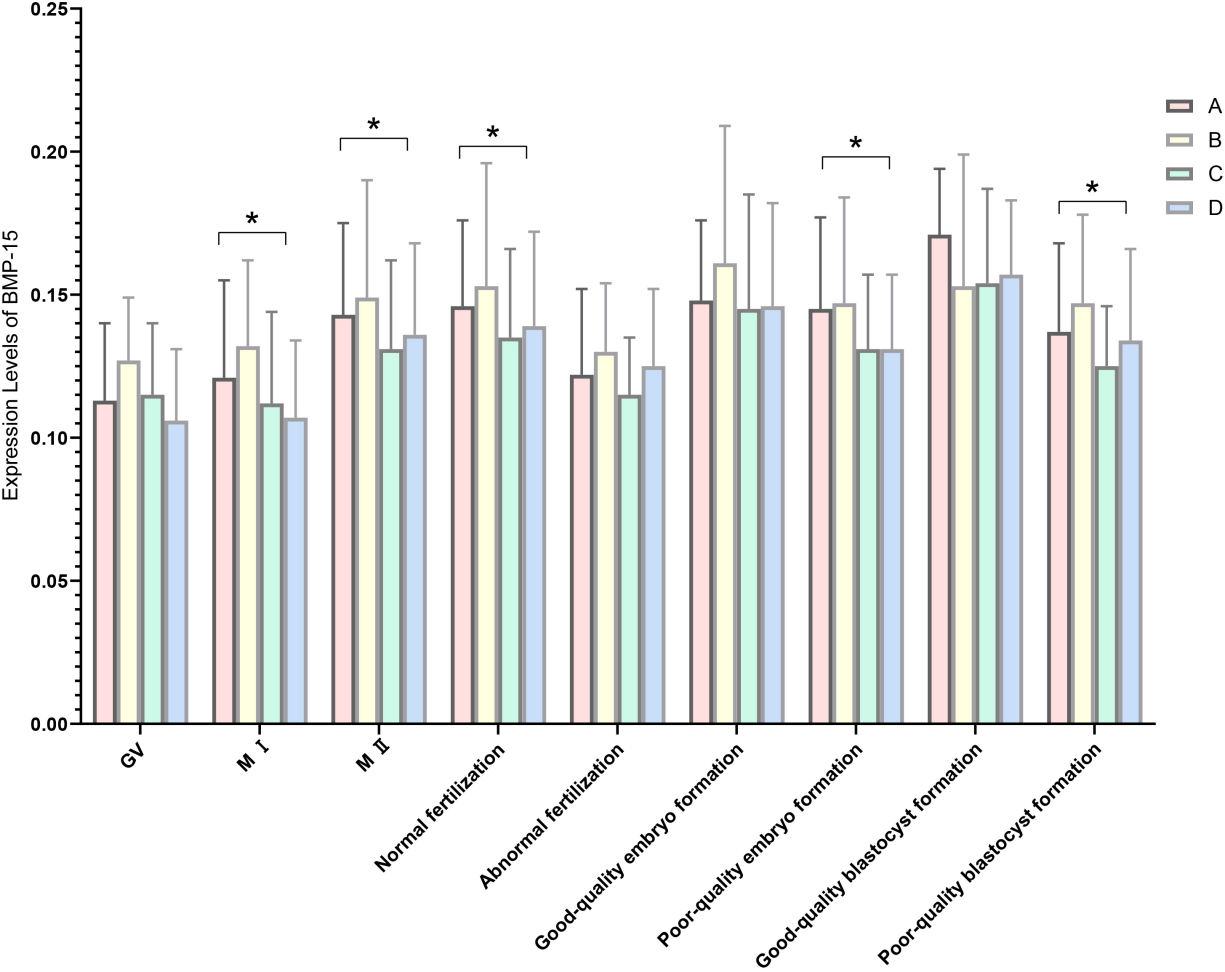
Expression levels of BMP-15 in four ovarian stimulation protocols. The different ovarian stimulation regiments are represented by different colored column. Group A is short-term long protocol during the luteal phase, displayed in red column; Group B is long-term long protocol during the follicular phase, displayed in yellow column; Group C is micro-stimulation protocol, displayed in green column; Group D is antagonist protocol, displayed in blue column. The Y-axis represents the mRNA expression level of BMP-15 obtained by RT-qpcr, n=3, * indicates p < 0.05. The X-axis indicates the different stages of RT-qpcr detection.

There were statistically significant differences in the relative expression of BMP-15 during the fertilization stage between the luteal phase standard long protocol and follicular phase long protocol (P = 0.007). During the cleavage stage, statistically significant differences were found between the luteal phase standard long protocol and both the microstimulation protocol and antagonist protocol (P = 0.033, P = 0.035), as well as between the follicular phase long protocol and microstimulation protocol (P = 0.034) (**Figure 3**, **Table 8**). During the blastocyst formation stage, expression levels differed significantly between the luteal phase standard long protocol and both the microstimulation protocol and antagonist protocol (P = 0.001, P = 0.013) (**Tables 1**, **7** and **8** for details).

**Table 8:** Expression levels of BMP-15 in four ovarian stimulation protocols ( $\bar{x}$ ± s).

Group	A group	B group	C group	D group	P Value
GV	0.113 ± 0.027	0.127 ± 0.022	0.115 ± 0.025	0.106 ± 0.025	0.156
MI	0.121 ± 0.034^a^	0.132 ± 0.030	0.112 ± 0.032^a^	0.107 ± 0.027^a^	0.001
MII	0.143 ± 0.032^a^	0.149 ± 0.041	0.131 ± 0.031^a^	0.136 ± 0.032	0.001
Normal fertilization	0.146 ± 0.030^b^	0.153 ± 0.043	0.135 ± 0.031	0.139 ± 0.033	0.003
Abormal fertilization	0.122 ± 0.030	0.130 ± 0.024	0.115 ± 0.020	0.125 ± 0.027	0.465
Good-quality embryo formation	0.148 ± 0.028	0.161 ± 0.048	0.145 ± 0.040	0.146 ± 0.036	0.144
Poor-quality embryo formation	0.145 ± 0.032^b^	0.147 ± 0.037	0.131 ± 0.026	0.131 ± 0.026^a^	0.032
Good-quality blastocyst formation	0.171 ± 0.023	0.153 ± 0.046	0.154 ± 0.033	0.157 ± 0.026	0.363
Poor-quality blastocyst formation	0.137 ± 0.031	0.147 ± 0.031	0.125 ± 0.021^a^	0.134 ± 0.032^a^	0.008

^a^indicates a statistical difference compared to group B, b:indicates a statistical difference compared to group C.

## 4 Discussion

This study aimed to investigate the expression levels of GDF-9 and BMP-15 across different ovarian stimulation protocols and their potential impact on oocyte maturation and developmental potential. Our results demonstrate that GDF-9 and BMP-15 exhibit distinct expression patterns depending on the ovarian stimulation protocol, and these patterns appear to be closely associated with oocyte maturation and embryo development. However, there are several limitations to this study, and the findings should be interpreted within the context of these shortcomings.

Our results indicate that the expression of GDF-9 and BMP-15 is substantially higher in MII oocytes compared with GV and MIical difference compared to gro oocytes, which is consistent with findings from previous studies (18, 19). Additionally, we observed that normal fertilization groups exhibited higher levels of GDF-9 and BMP-15 expression than abnormal fertilization groups, aligning with the established role of these molecules in folliculogenesis and oocyte maturation (20). Given that GDF-9 and BMP-15 regulate granulosa cell function, follicle development and oocyte quality (21), their expression could serve as a valuable biomarker for assessing oocyte developmental competence.

Despite these substantial findings, the molecular mechanisms underlying the regulation of these molecules in different ovarian stimulation protocols remain unclear. Future studies utilizing gene-editing techniques or animal models are warranted to elucidate how these molecules specifically influence oocyte maturation and embryo development in response to varying ovarian stimulation conditions (22).

Although this study provides valuable insights into the role of GDF-9 and BMP-15 in oocyte development, several limitations must be considered:

1. Sample Size and Diversity: Although the results are statistically significant within the sample size used, the overall sample size remains relatively small, particularly in the abnormal fertilization and poor-quality blastocyst formation groups. Expanding the sample size and including a more diverse cohort – encompassing patients of different age groups, ovarian reserves and underlying conditions – would enhance the generalizability of our findings (22).
2. Limited Range of Ovarian Stimulation Protocols: This study focused on four commonly used ovarian stimulation protocols (A, B, C, D). Expanding the analysis to include additional stimulation regimens and patients with varying ovarian responses (e.g. poor responders) would help determine whether GDF-9 and BMP-15 serve as universal biomarkers across different stimulation protocols (19, 21, 23).
3. Short-Term Follow-Up: The study primarily assessed oocyte maturation and early embryo development. Conducting a long-term follow-up to evaluate clinical pregnancy rates and live birth outcomes in relation to GDF-9 and BMP-15 expression would provide a clearer understanding of the role these molecules play in successful IVF outcomes (24, 25).
4. Mechanistic Insights: Although this study analyzed the expression levels of GDF-9 and BMP-15, the specific molecular pathways through which these factors regulate oocyte maturation and embryo development were not explored. Future research should investigate the underlying signaling pathways, particularly the TGF-β and SMAD pathways, which are known to be critical in ovarian function (21, 26).
5. Method of cumulus cells isolation: Although the cumulus cell isolation method follows the clinical practice, the cell heterogeneity and operation standardization still need to be optimized. Nevertheless, we have ensured internal consistency of the data through strict experimental procedures such as harmonization of enzymatic hydrolysis times and standardization of mechanical stripping.
6. Single center: The single-center design may limit the external validity of the results, and it is necessary to expand the sample diversity through multi-center cooperation in the future. Follow-up studies will combine single-cell techniques with multicenter cohorts to further validate the potential of GDF-9/BMP-15 as a marker of efficacy for ovarian stimulation.

Our findings align with previous studies demonstrating the involvement of GDF-9 and BMP-15 in oocyte maturation and embryo quality (27, 28). However, unlike some studies, we did not observe substantial differences in BMP-15 expression between certain groups, which may be attributed to variations in sample size or experimental conditions (29). This underscores the need for further research with larger sample sizes and a broader range of ovarian stimulation protocols to validate the role of these molecules in oocyte maturation.

Furthermore, the expression patterns of GDF-9 and BMP-15 in our study were consistent with the known influence of the TGF-β superfamily on follicular growth and oocyte quality (21, 26). Although the role of these growth factors in regulating granulosa cell proliferation and oocyte competence is well documented (30, 31), the precise mechanisms by which GDF-9 and BMP-15 mediate their effects in response to different ovarian stimulation protocols remain unclear.

Building on the limitations and findings of this study, several key research directions should be pursued. First, increasing the sample size and incorporating a more diverse cohort are essential to enhance the generalizability of our findings and strengthen the robustness of the results. Additionally, exploring different ovarian stimulation protocols, including newer approaches such as dual stimulation, could provide valuable insights into how GDF-9 and BMP-15 influence oocyte maturation and embryo development across varied patient populations.

Furthermore, future research should investigate the molecular mechanisms regulating GDF-9 and BMP-15 during oocyte maturation. Advanced techniques such as *CRISPR-Cas9* gene editing and RNA sequencing could offer a deeper understanding of the specific signaling pathways involved. Finally, long-term outcome studies are crucial for assessing clinical pregnancy rates and live birth outcomes in relation to GDF-9 and BMP-15 expression. These studies would help establish their predictive value as biomarkers for IVF success, ultimately contributing to more personalized fertility treatments.

## 5 Conclusion

In conclusion, this study offers valuable insights into the expression patterns of GDF-9 and BMP-15 in response to different ovarian stimulation protocols. Our findings suggest that GDF-9 and BMP-15 play a crucial role in regulating oocyte maturation and embryo development. However, the study’s limitations, including sample size, protocol diversity and lack of long-term follow-up, highlight the need for further investigation. Addressing these limitations in future research could help validate the role of these molecules as biomarkers for improving IVF outcomes and contribute to more personalized treatment strategies in assisted reproductive technology.

## Data Availability

The original contributions presented in the study are included in the article/supplementary material. Further inquiries can be directed to the corresponding author/s.
